# The minichromosome maintenance complex drives esophageal basal zone hyperplasia

**DOI:** 10.1172/jci.insight.172143

**Published:** 2023-09-08

**Authors:** Mark Rochman, Yrina Rochman, Julie M. Caldwell, Lydia E. Mack, John A. Besse, Nathan P. Manes, Sung Hwan Yoon, Tetsuo Shoda, Aleksandra Nita-Lazar, Marc E. Rothenberg

**Affiliations:** 1 Division of Allergy and Immunology, Cincinnati Children’s Hospital Medical Center, Department of Pediatrics, University of Cincinnati College of Medicine, Cincinnati, Ohio, USA.; 2 Functional Cellular Networks Section, Laboratory of Immune System Biology, National Institute of Allergy and Infectious Diseases, NIH, Bethesda, Maryland, USA.

**Keywords:** Gastroenterology, Immunology, Allergy, Proteomics

## Abstract

Eosinophilic esophagitis (EoE) is a chronic gastrointestinal disorder characterized by food antigen–driven eosinophilic inflammation and hyperproliferation of esophageal mucosa. By utilizing a large-scale, proteomic screen of esophageal biopsies, we aimed to uncover molecular drivers of the disease. Proteomic analysis by liquid chromatography–tandem mass spectrometry identified 402 differentially expressed proteins (DEPs) that correlated with the EoE transcriptome. Immune cell–related proteins were among the most highly upregulated DEPs in EoE compared with controls, whereas proteins linked to epithelial differentiation were primarily downregulated. Notably, in the inflamed esophageal tissue, all 6 subunits of the minichromosome maintenance (MCM) complex, a DNA helicase essential for genomic DNA replication, were significantly upregulated at the gene and protein levels. Furthermore, treating esophageal epithelial cells with a known inhibitor of the MCM complex (ciprofloxacin) blocked esophageal epithelial proliferation. In a murine model of EoE driven by overexpression of IL-13, ciprofloxacin treatment decreased basal zone thickness and reduced dilated intercellular spaces by blocking the transition of epithelial cells through the S-phase of the cell cycle. Collectively, a broad-spectrum proteomic screen has identified the involvement of the MCM complex in EoE and has highlighted MCM inhibitors as potential therapeutic agents for the disease.

## Graphical abstract

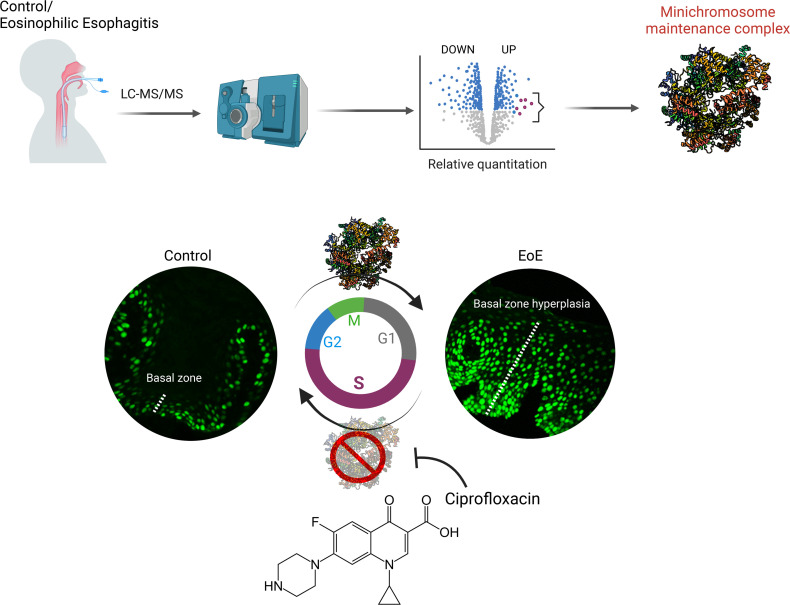

## Introduction

Eosinophilic esophagitis (EoE) is a chronic, allergic gastrointestinal disorder characterized by food antigen–driven eosinophilic inflammation of esophageal mucosa resulting in a damaged esophageal lining and leading to a variety of symptoms (e.g., failure to thrive, chest and abdominal pain, persistent heartburn, vomiting, dysphagia, food impactions). The inflammation in EoE is associated with the accumulation of immune cells, such as eosinophils, T cells, and mast cells, and profound changes in the esophageal epithelium, including basal zone hyperplasia (BZH) and dilated intercellular spaces (DIS) (1, 2).

BZH is a hallmark of EoE and is highly correlated with eosinophil and mast cell infiltration into inflamed tissue (3, 4). Notably, incorporating the degree of BZH into histologic assessments improves the classification of EoE disease severity (5, 6). Furthermore, although BZH is responsive to swallowed glucocorticoid treatment, it may persist during disease remission, and its expression correlates with persistent symptoms (7, 8). BZH is likely driven by the transcriptional response of the epithelium to the pro-atopy cytokine IL-13, which is corroborated by in vitro studies and in vivo murine models of EoE (9–11), and importantly by the clinical benefit of blocking IL-13 in patients (12–14). Functionally, proliferating epithelial cells uniquely contribute to disease pathogenesis by dysregulating a specific set of genes enriched in peptidase activity and other processes (15, 16), collectively suggesting that targeting BZH could potentially lead to better treatment strategies for EoE.

Large-scale proteomic analyses are becoming increasingly informative in allergic conditions, including asthma, allergic rhinitis, and atopic dermatitis (AD), providing insight into the underlying mechanisms of these diseases and identifying potential therapeutic targets (17–19). A recent proteomics analysis of esophageal fibroblasts from patients with active EoE explored the mechanisms of fibrosis (20). However, the comprehensive proteomic signature of esophageal tissue in EoE has not been reported. Herein, we employed liquid chromatography–tandem mass spectrometry (LC–MS/MS) to perform a large-scale proteomic analysis of esophageal biopsies from individuals with and without EoE.

We identified 402 differentially expressed proteins (DEPs) that were highly correlated with the esophageal transcriptome of EoE. Proteins related to eosinophils and other immune cells were highly upregulated in EoE compared with control biopsies, whereas proteins linked to epithelial differentiation were primarily downregulated. Additionally, all 6 subunits of the minichromosome maintenance (MCM) complex were significantly upregulated in the inflamed esophageal tissue at the gene and protein levels. The MCM complex is a double-hexamer ring structure composed of 6 MCM proteins, MCM2–MCM7, and its expression is tightly regulated at both the initiation and elongation stages of eukaryotic DNA replication (21). Elevated expression of MCM subunits is associated with cell transformation, whereas MCM subunit downregulation and cleavage are linked to reduced growth potential and induction of senescence in the epithelium, including in esophageal epithelial cells (22–24). Treating esophageal epithelial cells with the MCM complex inhibitor ciprofloxacin (25) reduced epithelial proliferation in vitro and improved histopathologic features in a murine model of EoE. Collectively, through comprehensive proteomic analysis, we identify in situ upregulation of the MCM complex in EoE, demonstrate that inhibiting this complex with ciprofloxacin counteracts BZH, and propose that ciprofloxacin and other MCM inhibitors may be therapeutic for EoE.

## Results

### EoE proteomic signature.

**Figure 1:**
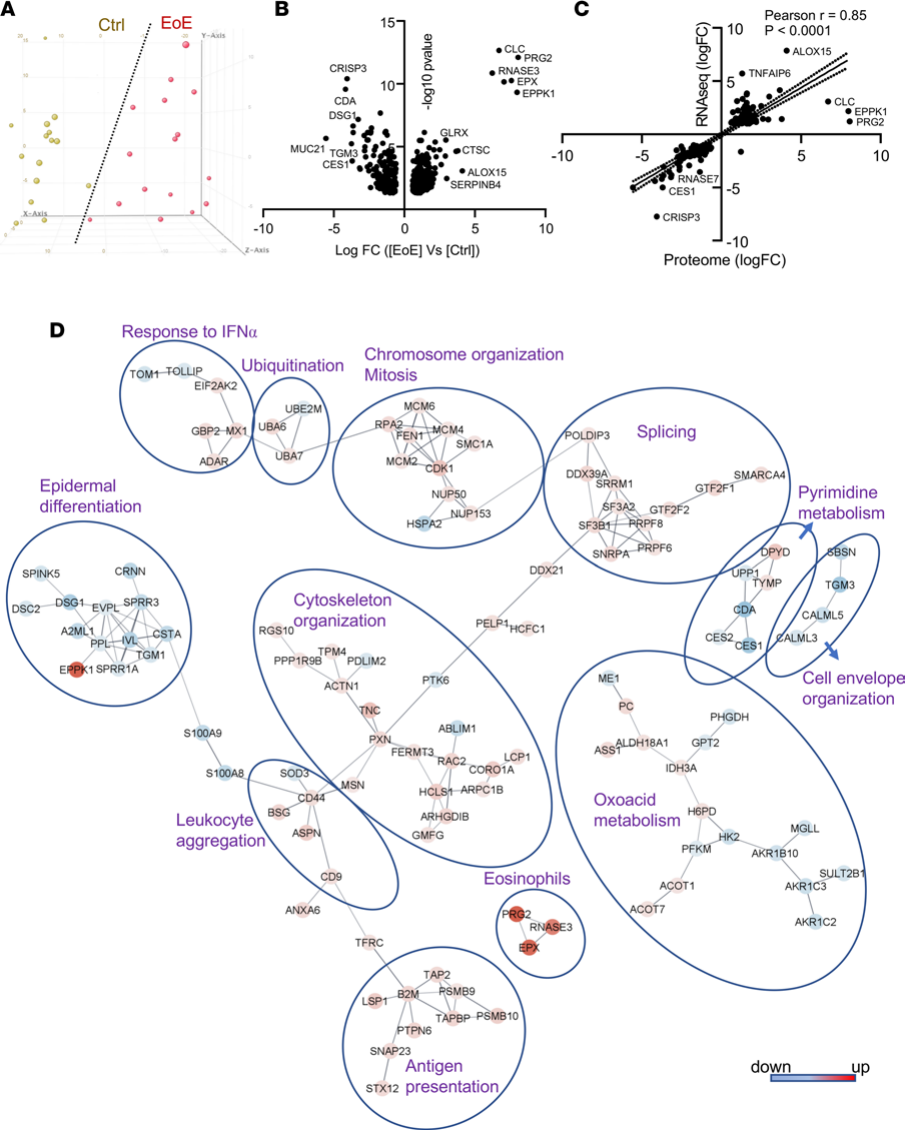
EoE proteomics signature. (**A**) Principal component analysis of the results of mass spectrometry results performed on the esophageal biopsies from the patients with active eosinophilic esophagitis (EoE, *n* = 16) and control patients having 0 eosinophils per high-power field (Ctrl, *n* = 15). (**B**) Volcano plot shows significantly differentially expressed proteins (DEPs; FDR < 0.05, fold change [FC] ≥ 1.5) between esophageal biopsies from the patients with active EoE and controls. The most highly DEPs are indicated. (**C**) Pearson’s correlation of EoE proteome and transcriptome (67). The most highly upregulated and downregulated genes are indicated. (**D**) EoE proteome is shown as DEPs (FDR < 0.05, FC > 2; individual nodes) colored by the FC compared with controls. The most significantly enriched biological processes for the groups of interacting proteins are indicated. Functional analysis was performed with the ToppGene suite (68).

**Figure 2:**
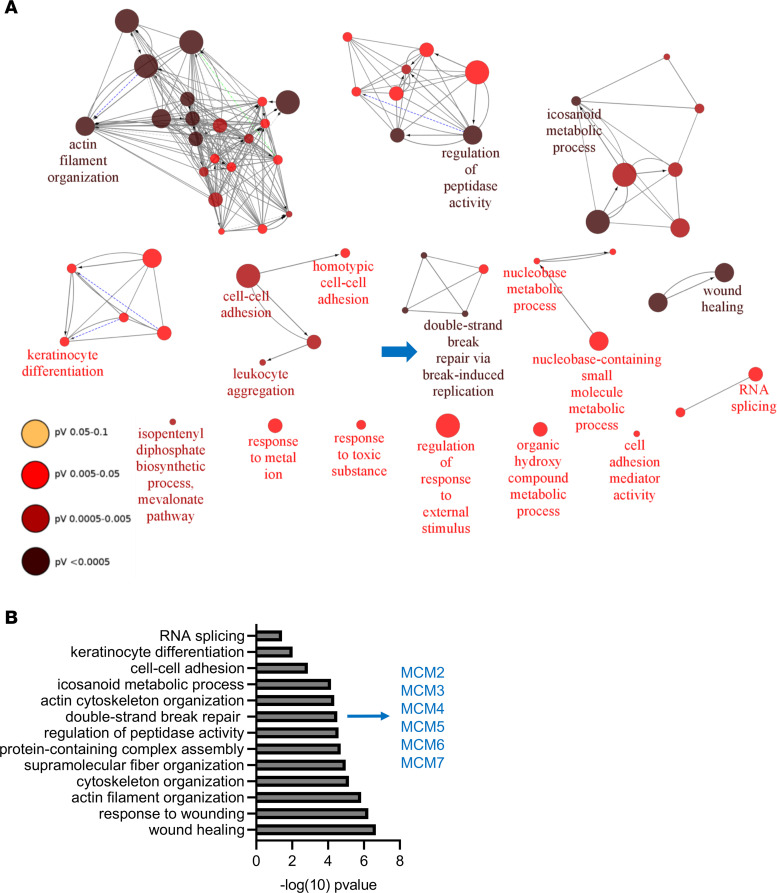
Functional characterization of EoE proteome. (**A**) The Gene Ontology (GO) functional network of the biological processes in the EoE proteome was generated by the ClueGo app in Cytoscape. Only the significantly enriched biological processes are shown (*P*_adj_ < 0.05), and individual nodes are colored by the *P* value (pV). The leading GO term for each group is indicated. (**B**) The graph shows the most significant biological processes enriched in the EoE proteome. Minichromosome maintenance (MCM) complex proteins represent the double-strand break repair term (indicated with the blue arrow in **A**).

We sought to identify the proteomic signature of esophageal tissue in patients with active EoE. We compared the biopsy proteomes of patients with EoE and controls using LC–MS/MS. Active EoE was defined as 15 or more eosinophils per high-power field (HPF) in the biopsy, and controls included both individuals with no history of EoE and patients with a history of EoE with no tissue-infiltrating eosinophils at the time of the biopsy (Supplemental Table 1; supplemental material available online with this article; https://doi.org/10.1172/jci.insight.172143DS1). A total of 2,203 proteins were detected by LC–MS/MS following strict filter criteria (FDR < 0.01 at the peptide spectrum match, peptide, and protein levels; label-free quantification [LFQ] with a minimum ratio count of 2; and at least 2 unique peptides per protein; Supplemental Table 2). Of the 2,203 identified proteins, 402 were differentially expressed between groups (FDR < 0.05, fold change [FC] > 1.5; Supplemental Table 3). Principal component analysis revealed a distinct separation of control and active EoE biopsies (Figure 1A). Eosinophil proteins were upregulated, whereas epithelial proteins, such as transglutaminase 3 (TGM3), desmoglein 1 (DSG1), and mucin 21 (MUC21), were downregulated in EoE biopsies (Figure 1B). We noted that there was a loss of esophagus-enriched proteins in EoE, consistent with loss of epithelial differentiation and proteolytic imbalance, as has been reported previously (2). For example, 80 esophagus-enriched proteins were downregulated; only 9 proteins were upregulated, including serine protease inhibitors (SERPINs) and calpain 14 (CAPN14), the latter of which is genetically linked to the disease (Supplemental Figure 1) (26). The proteomic signature of EoE strongly correlated with the transcriptional response (Pearson’s *r* = 0.85, *P* < 0.0001; Figure 1C). Interaction network analysis of DEPs using the STRING database (27) with a “high confidence” score requirement of at least 0.7 revealed functional groups linked to epithelial differentiation, immune cell infiltration, antigen presentation, cytoskeleton organization, metabolic processes, splicing, and mitosis (Figure 1D). Functional analysis showed the highest enrichment of the biological processes related to wound healing, double-strand break repair, actin organization, and peptidase activity (Figure 2, A and B). Collectively, the proteomic signature of EoE revealed robust esophageal tissue response to the inflammatory cues accompanied by profound histopathologic changes, including immune cell infiltration, metabolic alterations, and epithelial responses, such as BZH.

Given the high prevalence of AD in patients with EoE and similarities in AD and EoE molecular signatures (28, 29), we compared the EoE proteome to the joint proteomic signature of the lesional skin biopsies derived from patients with AD in 4 independent studies analyzed by O-link proteomics and LC–MS/MS (total of 764 proteins) (30–33). This comparison showed an overlap of 47 proteins largely linked to epithelial differentiation and proteolysis, which were significantly enriched, indicating the critical contribution of these processes to the pathogenesis of both diseases (*P* = 0.0014, Fisher’s exact test; Supplemental Figure 2).

### MCM complex drives esophageal epithelial proliferation.

**Figure 3:**
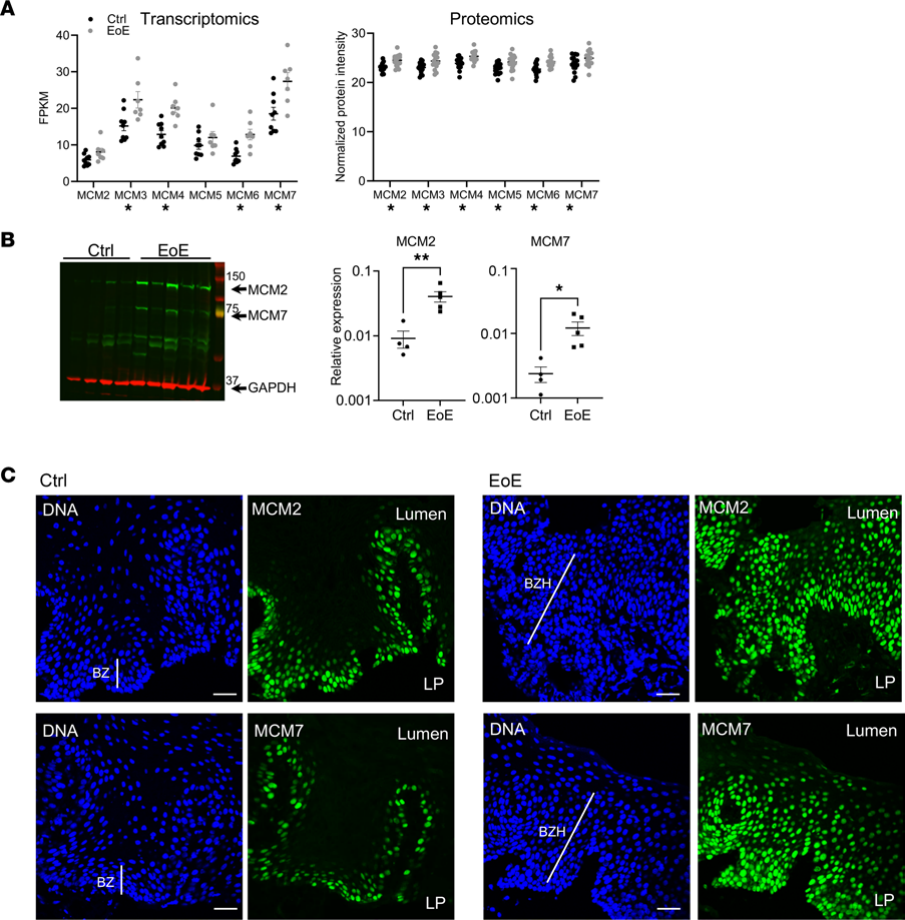
Expression of MCM proteins in human esophageal biopsies. (**A**) Expression of minichromosome maintenance (MCM) transcripts and proteins in the esophageal biopsies from control patients having 0 eosinophils per high-power field (Ctrl) and patients with active EoE was quantified by RNA sequencing (transcriptomics) (67) and mass spectrometry (proteomics). *Indicates significantly differentially expressed MCM genes (FDR < 0.05). (**B**) Expression of MCM2 and MCM7 in the esophageal biopsies from controls and patients with active EoE was assessed by Western blotting. The graphs show the quantification of the protein expression relative to the loading control (GAPDH). *P < 0.05, **P < 0.01 by unpaired, 2-tailed Student’s *t* test. In **A** and **B**, data are shown as mean ± SEM, with markers representing individual patients or controls. (**C**) Immunofluorescence images of MCM2 and MCM7 proteins (green) in the esophageal biopsies from controls and patients with active EoE. DNA was counterstained by Hoechst (blue). Scale bars: 100 μm. The white line represents the basal zone (BZ) on the control images and basal zone hyperplasia (BZH) on the active EoE images. LP, lamina propria.

Proteomics analysis found that mitosis and DNA replication pathways were enriched in EoE in part related to elevated expression of the MCM complex, a helicase composed of 6 proteins (MCM2–MCM7) responsible for DNA replication during cellular division (Figure 1D and Figure 2, A and B) (21). We therefore hypothesized that the MCM complex is a critical driver of the BZH that is characteristic of EoE. Elevated expression of the MCM complex was confirmed in patients with active EoE through transcriptome and proteome analyses (Figure 3A). Accordingly, Western blot analysis showed significantly higher expression of subunits 2 and 7 of the MCM complex (MCM2 and MCM7) in the biopsies from patients with active EoE compared with controls (Figure 3B). Immunofluorescence demonstrated that expression of both subunits was confined to nuclei of a few layers of the basal epithelium in control patients but was expanded to multiple epithelial layers in patients with active EoE, consistent with increased proliferation of the basal zone cells (Figure 3C).

**Figure 4:**
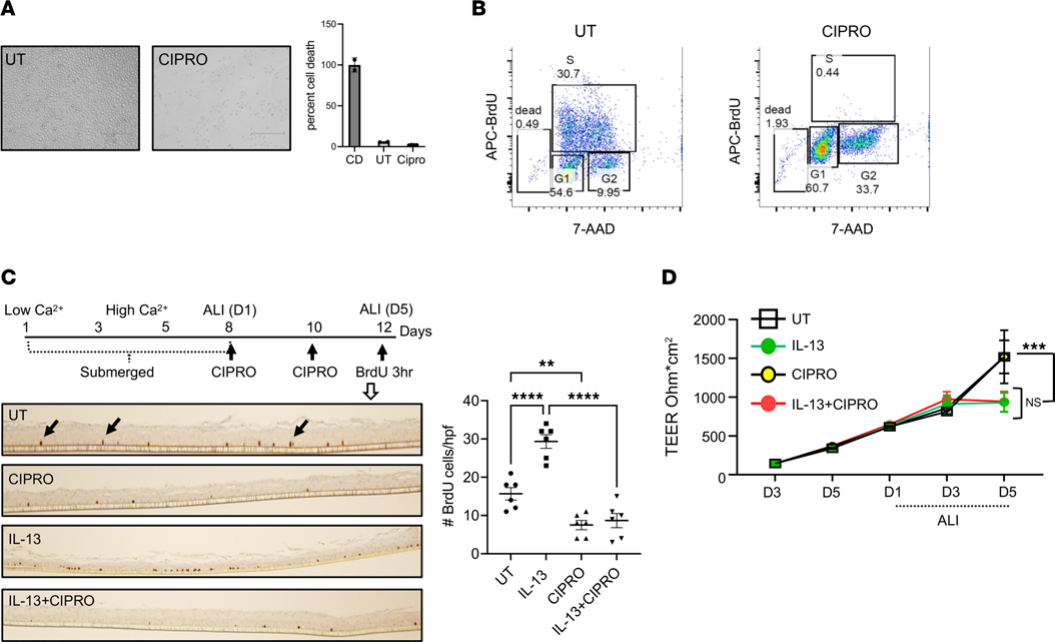
Inhibition of the MCM complex in EPC2 esophageal epithelial cells. (**A**) Representative images of EPC2 cells either untreated (UT) or treated with ciprofloxacin (CIPRO) for 4 days. The graph shows cell death as measured by the lactate dehydrogenase (LDH) release cytotoxicity assay and presented as mean ± SD. CD, 100% cell death control (cells treated with 0.2% Triton X-100). (**B**) Representative flow cytometry plots for the cell cycle analysis of EPC2 cells either untreated (UT) or treated with ciprofloxacin (CIPRO) for 4 days. The percentage of cells in each cell cycle phase is indicated. (**C**) A schematic outline of the differentiation protocol for EPC2 cells grown at the air-liquid interface (ALI), treated with ciprofloxacin and/or IL-13, and labeled with BrdU. Representative images of BrdU-positive cells, with examples of dark brown nuclei indicated by arrows. The graph shows the quantification (mean ± SEM) of the BrdU-positive cells in a high-power field (HPF); each marker represents 1 HPF for 3 independent experiments. (**D**) Transepithelial electrical resistance (TEER) was measured in the EPC2 cells differentiated at the ALI and treated as indicated. The combined data from 3 independent experiments performed in triplicate are presented as mean ± SEM. **P < 0.01; ***P < 0.001; ****P < 0.0001 by 1-way ANOVA with Holm-Šidák correction (**C**) or 2-way ANOVA with Holm-Šidák correction (**D**). NS, not significant.

We aimed to investigate the role of the MCM in mediating BZH-like responses. We focused on inhibiting the MCM complex with ciprofloxacin, which is known to block the helicase activity of the MCM complex (25). To this end, we examined the immortalized esophageal epithelial cell line EPC2, which has been widely used to model epithelial properties of the homeostatic and diseased human esophagus. Exposing EPC2 cells grown in a monolayer to ciprofloxacin led to cell cycle arrest, as evidenced by a dramatic decrease in the total number of cells, primarily in actively proliferating cells (cells in the S-phase), without causing cell death (Figure 4, A and B). Epithelial cells grown at the air-liquid interface (ALI) represent a commonly used in vitro model for studying esophageal epithelial differentiation under homeostatic and inflamed conditions. Exposing the EPC2 cells grown at an ALI to the proallergic cytokine IL-13 results in increased proliferation of the basal cells and decreased transepithelial electrical resistance due to a barrier defect (34). Treating the ALI cultures with ciprofloxacin during differentiation led to decreased epithelial proliferation, both at baseline and following IL-13 exposure, without affecting epithelial barrier integrity (Figure 4, C and D).

**Figure 5:**
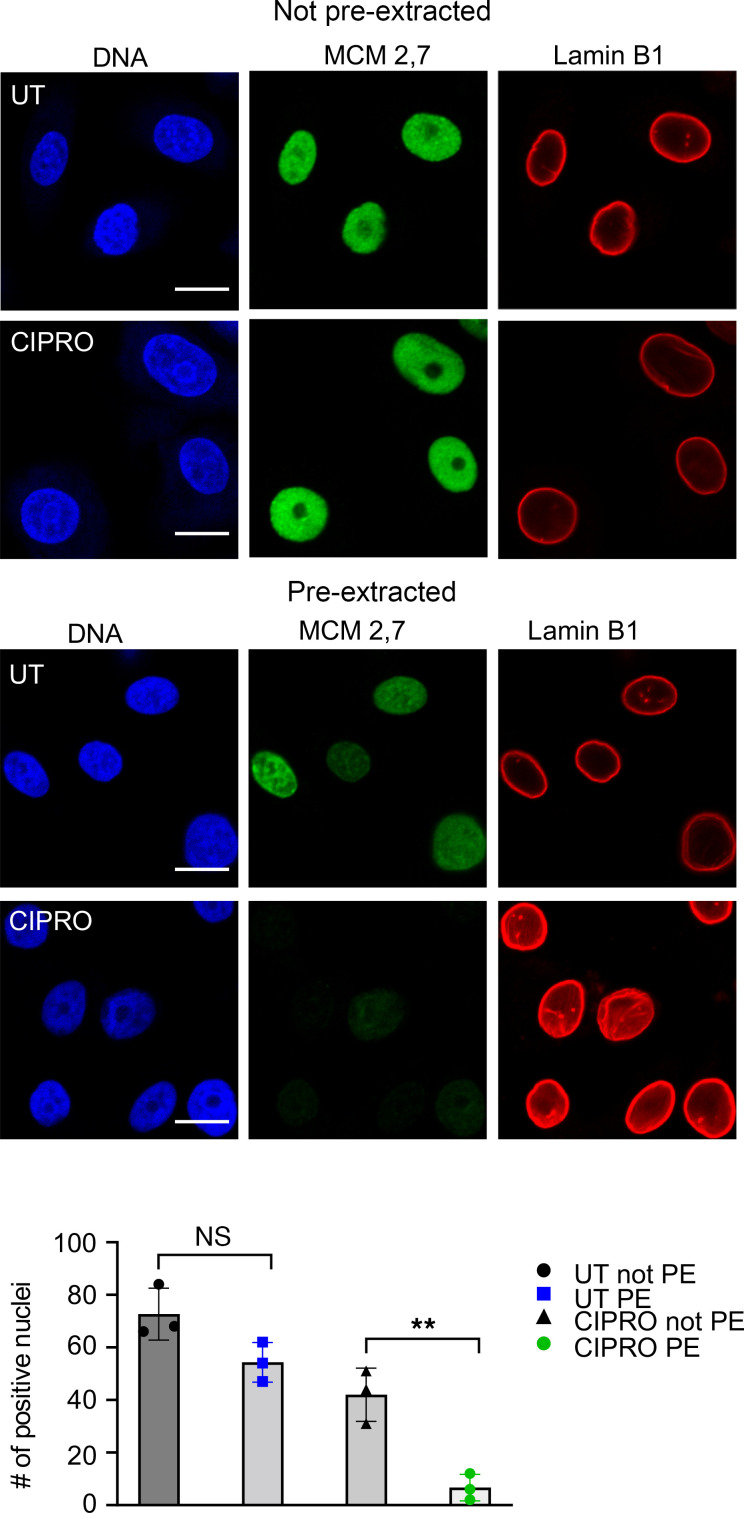
Effect of ciprofloxacin treatment on MCM extractability in EPC2 esophageal epithelial cells. Representative immunofluorescence images of untreated (UT) and ciprofloxacin-treated (CIPRO) EPC2 cells stained for MCM2 and MCM7 (green) and lamin B1 (red) and either not pre-extracted (PE) or pre-extracted with 0.2% Triton X-100 before fixing and staining. DNA was counterstained with Hoechst (blue). Scale bars: 10 μm. The graph shows the quantification of MCM2- and MCM7-positive cells in 3 independent images as mean ± SD. **P < 0.01 by unpaired, 2-tailed Student’s *t* test. NS, not significant.

We aimed to determine whether the decreased proliferation of the esophageal epithelial cells following ciprofloxacin treatment is mediated by its effect on the MCM complex. The activity of the MCM complex is regulated by a variety of mechanisms, the most critical of which is its binding to chromatin (35, 36). For nuclear proteins, extractability by detergents correlates with their ability to bind chromatin (37). Therefore, we assessed the detergent extractability of MCM2 and MCM7 subunits in untreated and ciprofloxacin-treated cells. Immunofluorescence showed a dramatic loss of both subunits but not lamin B1, a major structural component of the nuclear membrane, in the ciprofloxacin-treated cells following pre-extraction with Triton X-100 compared with untreated cells (Figure 5). These findings support the hypothesis that the MCM complex is a critical driver of esophageal epithelial proliferation in vitro.

### Ciprofloxacin treatment counteracts IL-13–mediated epithelial pathology in a murine model of EoE.

**Figure 6:**
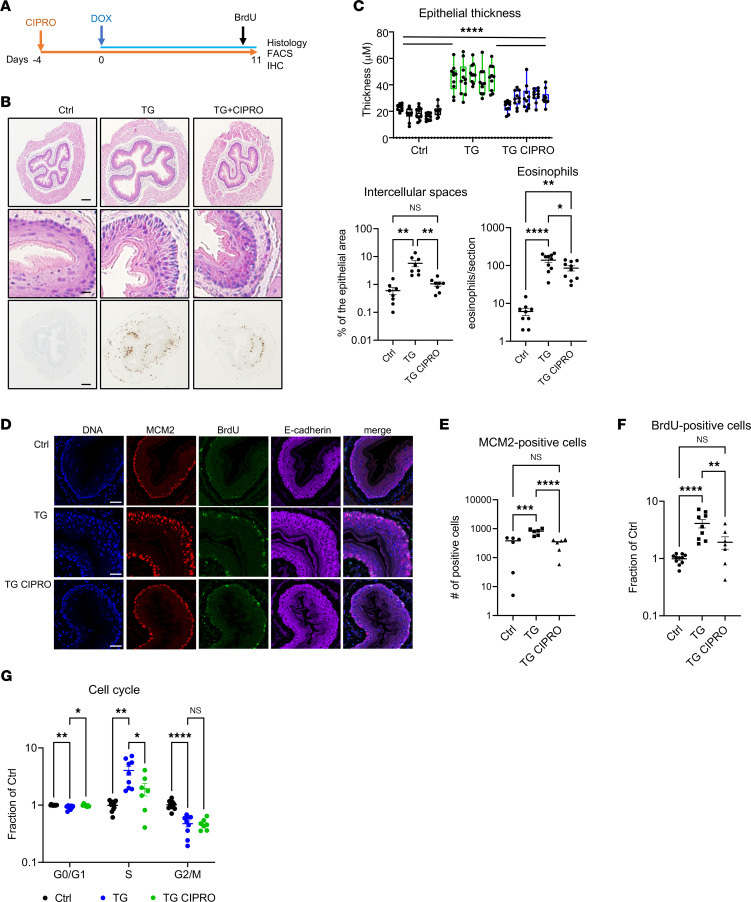
Effects of ciprofloxacin treatment on a mouse model of EoE. (**A**) A schematic outline of the mouse model of EoE, ciprofloxacin administration, and BrdU labeling in the CC10–IL-13 double transgenic mice. DOX, doxycycline. (**B**) Representative H&E images of the distal murine esophagus (top and middle images) and IHC images of the eosinophilic infiltration (bottom images) in the control (Ctrl), CC10–IL-13 double transgenic mice (TG), and CC10–IL-13 double transgenic mice treated with ciprofloxacin (TG + CIPRO). Scale bars: 100 μm (top and bottom images) and 50 μm (middle images). (**C**) Quantification of the epithelial thickness, intercellular spaces, and eosinophil infiltration in the esophagus as box-and-whisker plot. The box represents the 50th percentile of the data, whiskers show minimum and maximum values, and the line in the box represents the median. Each marker represents an individual measurement (see Methods). (**D**) Representative immunofluorescence images of the murine esophageal epithelium in the control, CC10–IL-13 double transgenic mice (TG), and CC10–IL-13 double transgenic mice treated with ciprofloxacin (TG + CIPRO). Scale bar: 50 μm. (**E**) Quantification of MCM2-positive cells in the esophageal epithelium as mean ± SEM. Each marker represents an individual section of the proximal and distal esophagus from 3 mice. (**F**) The fraction of actively proliferating BrdU-positive cells in the epithelium of the murine esophagus was determined by flow cytometry, normalized to the control mice, and is presented as mean ± SEM. Each marker represents an individual mouse. (**G**) Quantification of the esophageal epithelial cells in the phases of the cell cycle by flow cytometry as mean ± SEM. Each marker represents an individual mouse. *P < 0.05; **P < 0.01; ***P < 0.001; ****P < 0.0001 by 1 way ANOVA with Holm-Šidák correction (**C**, **E**, and **F**) or 2-way ANOVA with FDR correction by Benjamini, Krieger, and Yekutieli (**G**). NS, not significant.

We evaluated the effect of ciprofloxacin treatment on epithelial proliferation in a mouse model of EoE induced by transgenic overexpression of IL-13 (10). Mice were treated with ciprofloxacin for 2 weeks starting 4 days before IL-13 overexpression was induced by feeding animals with doxycycline. To measure actively proliferating cells, mice were injected with BrdU, a nucleoside analog that is incorporated into DNA during the S-phase of the cell cycle (Figure 6A) (38). Histologic evaluation showed that the doxycycline-treated IL-13–transgenic mice had increased epithelial thickness, cellular proliferation, and eosinophil accumulation compared with control mice. Ciprofloxacin treatment significantly decreased epithelial thickness and dilated intercellular spaces, reduced eosinophilic infiltration, and decreased the number of cells expressing MCM2 and BrdU compared with those of untreated mice (Figure 6, B–F). Cell cycle analysis of isolated esophageal epithelial cells (see Methods) showed that ciprofloxacin decreased the proportion of cells in the S-phase and increased the proportion of cells in the G_1_-phase compared with those of untreated mice. Both groups showed a decrease in the proportion of cells in the G_2_/M-phase (Figure 6G). Taken together, these findings indicate that ciprofloxacin treatment counteracts the IL-13–mediated epithelial pathology in a preclinical model of EoE.

## Discussion

In this study, we report on the proteomic signature of active EoE and propose a potential therapeutic benefit of inhibiting the MCM complex, a critical component of the proliferating epithelial cells in BZH. Previous studies that profiled the EoE proteome aimed to characterize extracellular matrix proteins secreted by fibroblasts (20), focused on cysteinyl-*S*-nitrosylated proteins in the esophageal biopsies (39), or developed a highly sensitive, antibody-like peptide–targeting method for detecting eosinophilic cationic protein in the mucus of patients with active EoE (40). Therefore, our work provides a unique perspective on the molecular mechanisms involved in EoE, complements previous EoE transcriptomics, and uncovers potential therapeutic targets for treating the disease.

Using a large-scale, highly sensitive proteomics approach (LC–MS/MS), we identified 402 DEPs in the esophageal biopsies from patients with active EoE compared with unaffected controls. Consistent with the histopathologic changes in EoE, the proteomic signature revealed immune infiltration into the inflamed esophageal tissue, with eosinophilic proteins being most highly upregulated. In contrast, proteins linked to epithelial differentiation, especially esophagus-enriched ones, were mostly downregulated, except for a few proteins linked to proteolytic activity (e.g., CAPN14 and SERPINs). A notable exception is epiplakin (EPPK1), a member of the plakin gene family that is enriched in the esophagus, which was highly elevated in EoE compared with controls. Plakins are large scaffold and adaptor proteins involved with signaling proteins that modulate cytoskeletal dynamics or cell migration and differentiation (41). Mutations conferring substantial risk for EoE have been identified in other plakin family members (e.g., desmoplakin [DSP] and periplakin [PPL]) that form desmosomes and are downregulated in EoE (42), collectively signifying the central role of the epithelial barrier and cytoskeletal organization in upholding the protective function of the esophageal epithelium. Moreover, since EPPK1 has been identified as a part of the EGF signaling pathway (43), its elevated expression in EoE may contribute to excessive epithelial proliferation leading to BZH.

Protein interaction analysis highlighted several functional pathways enriched in the EoE proteome with potential clinical utility. Antigen presentation is one such pathway and includes subunits of the immunoproteasome, proteasome subunit β type 9 (PSMB9) and 10 (PSMB10), being elevated in active disease (44). Notably, inhibiting the immunoproteasome, a specialized type of proteasome that is constitutively active in immune cells (44), showed beneficial effects in improving disease symptoms in various mouse models of inflammatory diseases, including allergic airway inflammation (45). Given the antiinflammatory potential and high selectivity demonstrated by immunoproteasome inhibitors in clinical trials for immune-mediated disorders (46), assessing these inhibitors for potential inclusion in the treatment options for EoE may offer benefits for patients. Furthermore, the high expression of the immunoproteasome in EoE suggests that general proteasome inhibitors may also prove beneficial. Inhibitors such as pentaerythritol tetrakis (PTTC) and ACU-D1 have been successfully tested in patients with skin inflammatory conditions like psoriasis and rosacea (47, 48).

Initial approaches to treat EoE include dietary restriction and/or medicine (e.g., proton pump inhibitors [PPIs], corticosteroids), but these therapies are only partially effective and are not well tolerated (49, 50). Although PPIs are recommended as a first-line treatment for EoE patients, the histopathological responses to PPIs vary greatly, and the long-term effects of their use, either alone or in combination with other therapies, remain unknown (51). Swallowed glucocorticoids are highly effective in improving the histological and clinical features of EoE; however, their adverse side effects and lack of FDA approval present significant drawbacks (51). Advanced treatment options include several biologics that target specific signaling pathways that trigger EoE (e.g., human anti–IL-4 receptor α antibody dupilumab, anti–IL-13 antibodies [e.g., cendakimab]) and eosinophilic infiltration of the tissue (e.g., Siglec-8 inhibitor lirentelimab, anti–IL-5RA antibody benralizumab, and anti–IL-5 antibodies mepolizumab and reslizumab). However, recent clinical trial data indicate that the latter agents, eosinophil-depleting antibodies, are not clinically effective (52–55). Although anti–IL-13 antibodies have shown the ability to reduce esophageal eosinophilia, improve the molecular signature of esophageal inflammation, and reverse epithelial-mesenchymal transition, their impact on the clinical manifestation of EoE remains to be determined (56). Recently, dupilumab, a monoclonal antibody targeting the IL-4 receptor α chain of the IL-4/13 receptor, received FDA approval as the only current therapy for EoE (54). Despite this success, numerous questions regarding optimal dosing, tolerability, and long-term effects still require further investigation.

Based on these findings, our study provides the rationale for the clinical development of a new EoE treatment approach by targeting a critical histologic feature of the disease that correlates with disease activity, BZH, utilizing specific inhibitors of the MCM complex that is required for cell proliferation. By utilizing the FDA-approved fluoroquinolone antibiotic ciprofloxacin in a preclinical model of EoE, we demonstrated the benefits of this approach, including decreased BZH and eosinophilic infiltration of the esophagus. Although we recognize that the systemic usage of ciprofloxacin or similar antiproliferative drugs to treat EoE may lead to side effects (57, 58), our findings can be considered as proof of principle for targeting BZH pathways in EoE.

**Figure 7:**
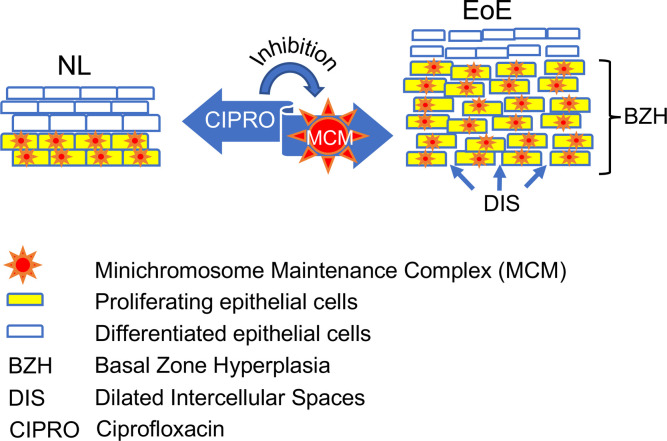
Schematic representation of beneficial ciprofloxacin effects in EoE. The inflamed tissue in eosinophilic esophagitis (EoE) is characterized by basal zone hyperplasia (BZH) and dilated intercellular spaces (DIS). These histopathologic features are driven by the activity of the minichromosome maintenance complex (MCM) in the proliferating cells. Ciprofloxacin (CIPRO) blocks MCM, leading to a decrease in BZH and DIS. NL, normal tissue.

In summary, a broad-spectrum proteomic screen by LS–MS/MS has led to the identification of the MCM complex’s involvement in EoE. Functional studies substantiated MCM involvement in esophageal BZH. Therefore, MCM inhibitors, such as ciprofloxacin, are now potential therapeutic agents for EoE (Figure 7). These findings demonstrate the potential value of unbiased proteomic analyses of eosinophilic diseases.

## Methods

### Participant inclusion criteria and biopsy sample acquisition

Biopsies were acquired from individuals who were having an endoscopy for EoE or related symptoms at Cincinnati Children’s Hospital Medical Center (CCHMC). Active EoE was defined by a histologic finding of 15 or more esophageal eosinophils per microscopic HPF with clinical symptoms. Controls included histologically normal individuals with no history of EoE and patients with a history of EoE with the endoscopy showing 0 eosinophils/HPF at the time of the biopsy. Individuals’ information was obtained from electronic medical records and research questionnaires. Patient data are summarized in Supplemental Table 1. Participants provided written informed consent for inclusion in an IRB-approved protocol.

### Antibodies

Primary antibodies were as follows: rabbit anti-MCM2 and anti-MCM7 (Cell Signaling Technology, catalog 3619 and 3735); goat anti–lamin B1 (Santa Cruz Biotechnology, catalog sc-6217); mouse anti-BrdU (Cell Signaling Technology, catalog 5292); mouse anti-GAPDH and anti-HSP90 (OriGene, catalog TA802519 and TA500494); mouse APC-conjugated anti-BrdU (BioLegend, catalog 364113); and goat anti–E-cadherin (R&D Systems, catalog AF648). 7-Amino-actinomycin D (7-AAD) was from BD Biosciences (catalog 559925). Hoechst 33342 Solution 20 mM was from Thermo Fisher Scientific (catalog 62249). Secondary IRDye-conjugated antibodies and Odyssey IR imager with Image Studio software were from LI-COR Biosciences.

### Proteomics of human esophageal biopsies

#### Sample collection.

Esophageal biopsies were sonicated for 4 minutes in 110 μL of 8 M urea with protease inhibitors in a microTUBE using an S220 focused ultrasonicator (Covaris, LLC). Sonication conditions were used as recommended by the manufacturer (10% output, 70 W peak incident power, 200 cycles per burst). Samples were spun down at 12,000*g* for 10 minutes, and the supernatant was collected for analysis. The bicinchoninic acid (BCA) protein assay was used to determine the protein concentration of each sample (Thermo Fisher Scientific, PI23227).

#### Digestion of proteins.

To digest proteins, 20 μg of each sample (protein mass) was resuspended in 100 μL of 6 M urea and 50 mM ammonium bicarbonate solution (pH 7.8). To reduce disulfide bonds, 6.7 μL of Tris buffer (pH 8.8) and 2.5 μL of 200 mM tris(2-carboxyethyl) phosphine were added, and the samples were incubated at 37°C for 1 hour. To alkylate cysteine residues, 20 μL of 200 mM iodoacetamide (IAM) was added, and the solution was incubated in the dark at room temperature for 1 hour. To quench unreacted IAM, 20 μL of 200 mM dithiothreitol (DTT) was added, and the samples were incubated at room temperature for 1 hour. To digest the samples, 800 μL of 25 mM ammonium bicarbonate, 200 μL of methanol, and 1 μg of sequencing-grade modified trypsin (Promega) were added, and the samples were incubated at 37°C for 18 hours. Sep-Pak C18 cartridges (Waters) were used for peptide sample cleaning and desalting following the manufacturer’s protocol.

#### MS.

An Orbitrap Eclipse (Thermo Fisher Scientific) coupled to an UltiMate 3000 (Thermo Fisher Scientific) was used for LC–MS/MS experiments. Two micrograms of each sample was injected for LC–MS/MS analysis. Peptides were trapped on an Acclaim C18 PepMap 100 trap column (5-μm particles, 100-Å pores, 300-μm i.d. × 5 mm, Thermo Fisher Scientific) and separated on a PepMap RSLC C18 column (2-μm particles, 100-Å pores, 75-μm i.d. × 50 cm, Thermo Fisher Scientific) at 40°C. The LC steps were 98% mobile phase A (0.1% v/v formic acid in H_2_O) and 2% mobile phase B (0.1% v/v formic acid in acetonitrile) from 0 to 5 minutes, 2% to 35% linear gradient of mobile phase B from 5 to 155 minutes, 35% to 85% linear gradient of mobile phase B from 155 to 157 minutes, 85% mobile phase B from 157 to 170 minutes, 85% to 2% linear gradient of mobile phase B from 170 to 172 minutes, and 2% mobile phase B from 172 to 190 minutes. Eluted peptides were ionized in positive ion polarity at a spraying voltage of 2.1 kV. MS1 full scans were recorded in the range of 375 to 1,500 *m*/*z* with a resolution of 120,000 at 200 *m*/*z* using the Orbitrap mass analyzer. Automatic gain control and maximum injection time were set to standard and auto, respectively. Top 3 seconds data-dependent acquisition mode was used to maximize the number of MS2 spectra from each duty cycle. Higher-energy collision-induced dissociation was used to fragment selected precursor ions with a normalized collision energy of 27. MS2 scans were recorded using an automatic scan range with a resolution of 15,000 at 200 *m*/*z* using the Orbitrap mass analyzer.

#### Initial analysis of MS results.

The RAW MS files were processed with MaxQuant (version 2.0.3.0, Max Planck Institute, Munich, Germany) (59) and searched with the Andromeda search engine (60) against a human UniProt FASTA database (61) (download date: September 27, 2021) supplemented with common contaminants and reverse sequences of all entries (62). The Andromeda search engine parameters were: type = standard; fixed modification = carbamidomethylation of cysteine; variable modifications = oxidation of methionine, acetylation of lysine, and acetylation of protein N-terminus; minimum peptide length = 7; and maximum missed cleavages = 2. The FDR was set to 0.01 at the peptide spectrum match, peptide, and protein levels. LFQ was performed with a minimum ratio count of 2. The “match between runs” function was used with a matching time window of 0.7 minutes and an alignment time window of 20 minutes. The default settings were applied for all the other parameters. The MS proteomics data have been deposited to the ProteomeXchange Consortium (63) via the PRIDE partner repository (64) with the data set identifier PXD040030.

### Analysis of the proteomics data

The MaxQuant LFQ values were used for the downstream analysis. Data analysis was performed using GeneSpring version 14.9 (Agilent Technologies). Median normalization was implemented, and DEPs were identified (*t* test with Benjamini-Hochberg FDR correction, FDR < 0.05, FC > 1.5). Hierarchical clustering was performed using the DEPs. Functional analysis was performed using Cytoscape by String and ClueGO applications (27, 65, 66). For the STRING analysis, a subset of the DEPs (FDR < 0.05, FC > 2) were used, and a stringency of 0.7 (described as “high confidence”) was applied to generate the connectome. The entire human genome was used as the reference gene set for the enrichment analyses. Correlation of the proteomics with the transcriptomics was performed using expression data from the EoE transcriptome (67).

### Cell culture and viability

The esophageal hTERT-immortalized human epithelial cell line EPC2 was a gift from Anil Rustgi (Columbia University, New York, New York, USA). Monolayer EPC2 cells were grown in keratinocyte serum-free media (KSFM) (Thermo Fisher Scientific, 17005042). For fractionation experiments, untreated or ciprofloxacin-treated cells were trypsinized and resuspended in 100 μL of PBS/0.2% Triton X-100 with protease inhibitors for 5 minutes at room temperature. Cells were spun at 300*g* for 5 minutes, and supernatants were collected. Pellets were washed once with 500 μL of PBS/0.2% Triton X-100. Pellets and supernatants were resuspended in lithium dodecyl sulfate (LDS) loading buffer and analyzed by Western blotting. For ALI culture, 150,000 EPC2 cells were grown to confluence while fully submerged in standard KSFM (0.09 mM CaCl_2_) on 0.4 mm pore size polyester permeable supports (Corning, 3470). Confluent monolayers were then switched to high-calcium KSFM (1.8 mM CaCl_2_) for an additional 5 days. To induce epithelial differentiation, the culture medium was removed from the inner chamber of the permeable support to expose the cells to the ALI. Where indicated, cultures were treated with ciprofloxacin, and BrdU was added to a final concentration of 3 mg/mL for 3 hours before fixation. Transepithelial electrical resistance was measured using an EVOM2 (World Precision Instruments). CyQUANT LDH Cytotoxicity Assay (Thermo Fisher Scientific, C20300) was used to measure cell viability per manufacture instructions.

### Immunofluorescence of cells and biopsies

For immunofluorescence, EPC2 cells were grown on μ-Slide 4 Well ibiTreat (Ibidi, 80426) at 50,000 cells per well. The next day, ciprofloxacin was added at a final concentration of 100 mg/mL for 48 hours. Cells in some wells were pre-extracted with PBS/0.2% Triton X-100 for 5 minutes at room temperature prior to fixation, washed once with PBS, fixed with 4% paraformaldehyde, and permeabilized with 0.5% Triton X-100 for 10 minutes. Blocking was performed in 10% goat serum for 30 minutes at room temperature, and the cells were incubated with the indicated primary antibodies at 1:250 dilution for 2 hours at room temperature. The secondary antibodies and Hoechst 33342 were added for 1 hour at room temperature at 1:500 dilution. For the immunofluorescent staining of the formalin-fixed, paraffin-embedded (FFPE) sections of biopsies and murine esophagi, antigen retrieval was performed using R-UNIVERSAL Epitope Recovery Buffer (EMS, 62719-20) in a pressure cooker for 15 minutes at 110°C. At least 3 biopsies from control patients and patients with active EoE were stained. The slides were blocked with 10% donkey serum. Primary antibodies were used at 1:200 dilution, and secondary antibodies at 1:500 dilution. Nuclei were counterstained with Hoechst 3342 (Thermo Fisher Scientific, H3570) at 1 mg/mL added together with the secondary antibodies. Imaging was performed with a Nikon A1 inverted confocal microscope in the Confocal Imaging Core at CCHMC.

### Western blotting

Proteins from control participants and patients with EoE were isolated by TRIzol and resuspended in 1× loading buffer comprising NuPAGE LDS Sample Buffer (4×) (Thermo Fisher Scientific, NP0007) premixed with radioimmunoprecipitation (RIPA) assay buffer (Thermo Fisher Scientific, 89900) to a final concentration of 1× and supplemented with 5% β-mercaptoethanol. Samples were sonicated with a probe sonicator 3 times for 10 seconds each with 30% output and boiled. Protein lysates were subjected to electrophoresis on a 4%–12% protein gel and probed with the indicated antibodies. Scanning was performed on an Odyssey CLx imager, and quantification of the signal was performed with Image Studio Lite (LI-COR Biosciences).

### Mouse model of EoE

Bitransgenic mice (CC10-iIL-13) were used for the murine model of EoE (10). Transgene expression was induced by feeding mice Mod RMH-1500 Auto with 0.0625% Doxycycline diet (Testdiet) for 10 days. Ciprofloxacin intraperitoneal injections (100 mg/kg/day) were administered daily starting 4 days before the introduction of DOX food throughout the experiment. Control animals received sterile water. Four hours before the termination of the experiment, all mice were injected with BrdU at 1 mg per mouse intraperitoneally. Three to 5 mice per treatment group were used in 3 independent experiments. Animals were housed under specific pathogen–free conditions per institutional guidelines.

### Collection, processing, and quantification of changes in the mouse esophagi

For histology and immunohistochemistry (IHC), esophagi were fixed in 10% formalin for 24 hours and processed by the Pathology Research Core at CCHMC to FFPE tissue blocks and 5-μm sections. BrdU-positive cells were detected by IHC. Esophageal eosinophils were detected using an immunohistochemical stain against the murine eosinophilic major basic protein (MBP) as reported previously (10). ImageJ (NIH) was used to quantify eosinophils, epithelial thickness, and dilated intercellular spaces. Eosinophils and MCM2-positive cells were quantified in the individual sections by setting up the threshold and using the “analyze particles” function on either the blue or red channel. Intercellular spaces were quantified using H&E sections as a percentage of the area outlined in the epithelial layer of the esophagus. Epithelial thickness was quantified using H&E sections with the “ROI management” tool from 10 random measurements per sample.

For cell cycle analysis, the outside muscle layer was mechanically removed from the esophagi by tweezers. Esophagi were washed in HBSS and treated with Dispase II (Thermo Fisher Scientific, 17105041) freshly dissolved in 2 mL of PBS/sample to 1.2 units/mL at 37°C for 45–60 minutes with gentle shaking. The epithelium was mechanically separated from the lamina propria under the microscope and submerged in 1 mL of 0.05% Trypsin-EDTA solution (Thermo Fisher Scienitific, 25300062). The epithelium was incubated for 10 minutes at 37°C with gentle shaking followed by vortexing for 10 seconds. Floating cells were collected in 8 mL of RPMI medium supplemented with 10% FCS, the epithelium was minced into small pieces, and incubation with trypsin was repeated. Epithelial cells were combined with previously collected cells in the RPMI 10% FCS medium and passed through a 70-μm cell strainer into 50-mL conical tubes. The strainers were rinsed with 10 mL of RPMI 10% FCS medium, and the cells were pelleted at 400*g* for 10 minutes at 4°C. Epithelial cells were processed for cell cycle analysis by flow cytometry according to the APC BrdU Kit manual (BD Biosciences, 552598).

### Cell cycle analysis by FACS

Data were acquired on an LSR Fortessa flow cytometer (BD Biosciences) and analyzed with FlowJo software. Cell doublets were excluded from the analysis.

### Statistics

Statistical analyses were performed with GraphPad Prism software version 9. Data are reported as mean ± SEM. For the comparison of 2 groups, statistical significance was determined by unpaired, 2-tailed Student’s *t* tests. For the comparison of more than 2 groups, either 1- or 2-way ANOVA as indicated in the figure legends with Holm-Šidák correction was used, and FDR less than 0.05 was considered significant.

### Study approval

Samples were obtained following informed consent under the auspices of the IRB of CCHMC (no. 2008-0090).

### Data availability

The MS proteomics data have been deposited to the ProteomeXchange Consortium (63) via the PRIDE partner repository (64) with the data set identifier PXD040030. Values for all data points shown in graphs and values behind any reported means are reported in the Supporting Data Values file.

## Author contributions

MR and MER designed the study. MR, YR, JMC, LEM, JAB, NPM, and SHY performed experiments. MR, YR, TS, NPM, and SHY analyzed the data. MR and MER wrote the manuscript. MER and ANL supervised the study. All authors reviewed and edited the manuscript.

## Supplementary Material

Supplemental data

Supplemental table 1

Supplemental table 2

Supplemental table 3

Supporting data values

## Version 1 07/25/2023

In-Press Preview

## Version 2 09/08/2023

Electronic publication
