# Supplementary material for: The minichromosome maintenance complex drives esophageal basal zone hyperplasia
Source: JCI Insight. 2023 Sep 8;8(17):e172143. doi: 10.1172/jci.insight.172143 (PMC10544209; doi:10.1172/jci.insight.172143)
Supplement: Supplemental data [file jciinsight-8-172143-s151.pdf]

A

GeneID	log (FC)	FDR
SERPINB4	3.01	0.003351164
MUC4	1.99	0.008203057
CAPN14	1.63	0.001317217
DUOXA1	1.58	0.001658379
DUOX1	1.51	0.000266283
EHD3	1.39	0.000126319
KLK7	1.00	0.04580813
SH3BP1	0.92	0.001551738
SERPINB2	0.76	0.037294094
TRIM29	-0.60	0.000595533
DSP	-0.63	0.000831728
HSPB1	-0.63	0.009846089
CBR3	-0.68	0.002854969
ANXA2	-0.69	0.004727191
LGALS7	-0.70	0.03490269
S100A14	-0.76	0.021431485
PPP1R13L	-0.87	0.001275085
PKP1	-0.88	2.32938E-05
C6orf132	-0.96	0.000943198
S100A16	-0.97	0.006526884
EPS8L2	-1.00	0.000144464
ADH7	-1.03	0.000180444
GBP6	-1.04	0.001871166
EVPL	-1.04	2.69655E-05
SCRIB	-1.05	0.023127137
MAPK13	-1.20	0.018760111
CLIC3	-1.20	3.92018E-05
SPRR1A	-1.21	0.006095917
PPL	-1.22	1.21795E-06
IL18	-1.28	3.52085E-05
CLCA4	-1.28	0.001440643
PRSS27	-1.30	0.019762246
KLK11	-1.33	0.005744916
TPRG1	-1.33	0.026035337
GLTP	-1.35	0.011944486
UPP1	-1.36	0.04351838
RNASE7	-1.36	0.009087686
CAPNS2	-1.37	0.009190067
KLK13	-1.37	0.006381359
DSC2	-1.37	7.5831E-07
SERPINB1	-1.37	0.000713884
ALDH3A1	-1.38	0.000159138
PTK6	-1.38	0.000817297
CALML3	-1.43	0.000262819
TGM1	-1.53	0.006095917
SPINK5	-1.55	0.016773922
SULT2B1	-1.56	0.022253204
FABP5	-1.57	0.000249112
EPS8L1	-1.68	6.13868E-05
NCCRP1	-1.69	0.002825466
CSTB	-1.71	2.13453E-08
AKR1B10	-1.75	0.005555187
HOPX	-1.76	0.00148852
TTC22	-1.86	0.003480898
DHRS1	-1.90	0.003024803
GDPD3	-1.91	0.001795998
DAPL1	-1.98	0.008579951
ECM1	-2.02	9.94151E-06
VSIG10L	-2.12	1.84262E-05
SPRR3	-2.18	0.000637926
CYSRT1	-2.19	0.001954498
S100A9	-2.33	3.33048E-07
S100A8	-2.37	6.62578E-07
A2ML1	-2.39	7.98934E-07
SBSN	-2.40	0.018006923
CSTA	-2.42	0.000267202
TMPRSS11E	-2.52	0.000852853
TP53I3	-2.57	7.41114E-07
CRNN	-2.60	7.5831E-07
PADI1	-2.64	2.94524E-06
TMPRSS11B	-2.74	6.55682E-05
IL36A	-2.79	1.04486E-05
IVL	-2.82	2.15647E-05
FAM25G	-2.85	1.31469E-06
SLURP1	-3.14	0.000626909
CRCT1	-3.18	0.000495562
ENDOU	-3.64	2.24827E-07
TGM3	-3.75	5.86835E-06
CDA	-4.17	2.70305E-10
MUC21	-5.56	2.20566E-06

B

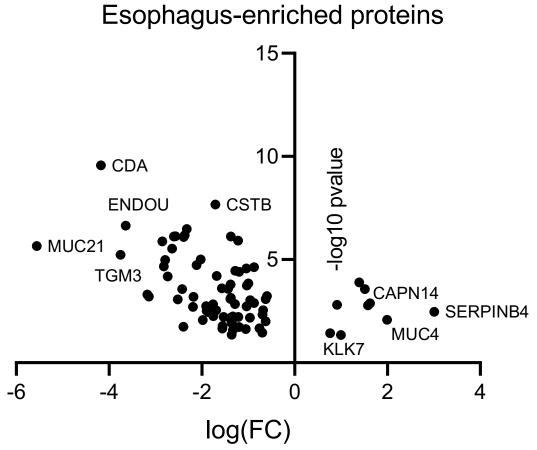

Supplementary Fig.1 A, The list of esophagus-enriched proteins identified in the EoE proteome. log(FC) represents a log2-transformed expression difference between EoE and control samples. B, Volcano plot of the esophagus-enriched proteins. Most differentially expressed proteins (DEPs) are labeled.

A

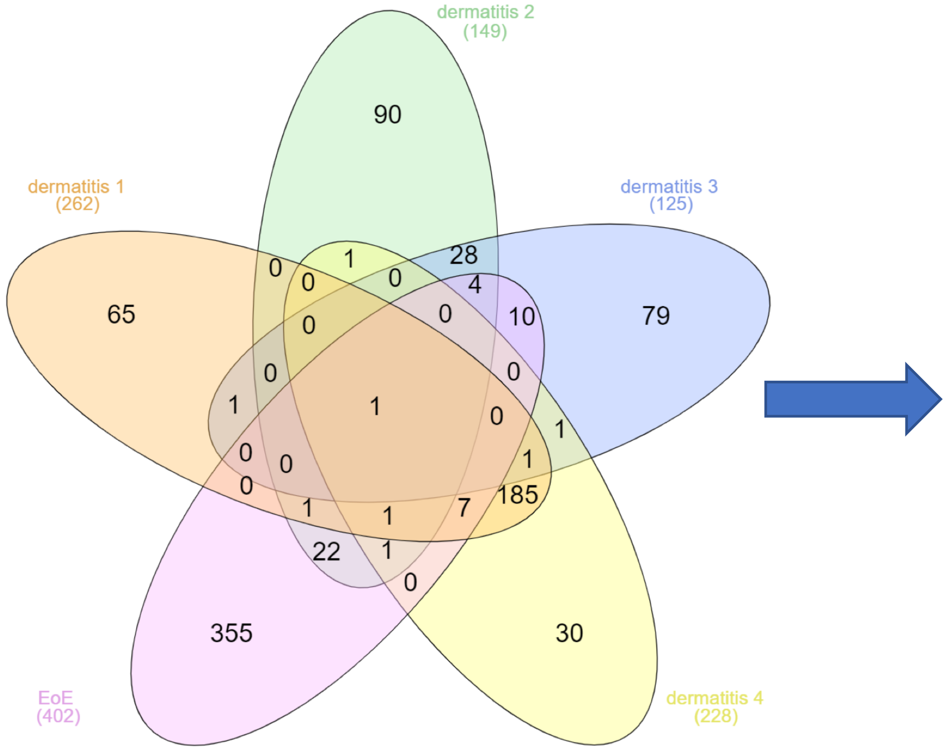

A2ML1
ACPP
ANXA2
BLMH
CALML5
CAPNS2
CDH3
CRNN
CSTA
CSTB
CTSC
CTSD
CTSL
DSG1
DSP
ECM1
EPPK1
GGH
GRN
HEXB
HSPB1
IL18
IMPA2
KLK11
KLK7
LGALS7
ME1
NCCRP1
PADI1
PARP1
PGRMC2
PRSS27
RNASE7
RNASET2
S100A14
S100A8
S100A9
SBSN
SERPINB1
SERPINB2
SLURP1
SPINK5
SPRR1A
TFRC
TGM1
TGM3
TPM4
VWA5A

B

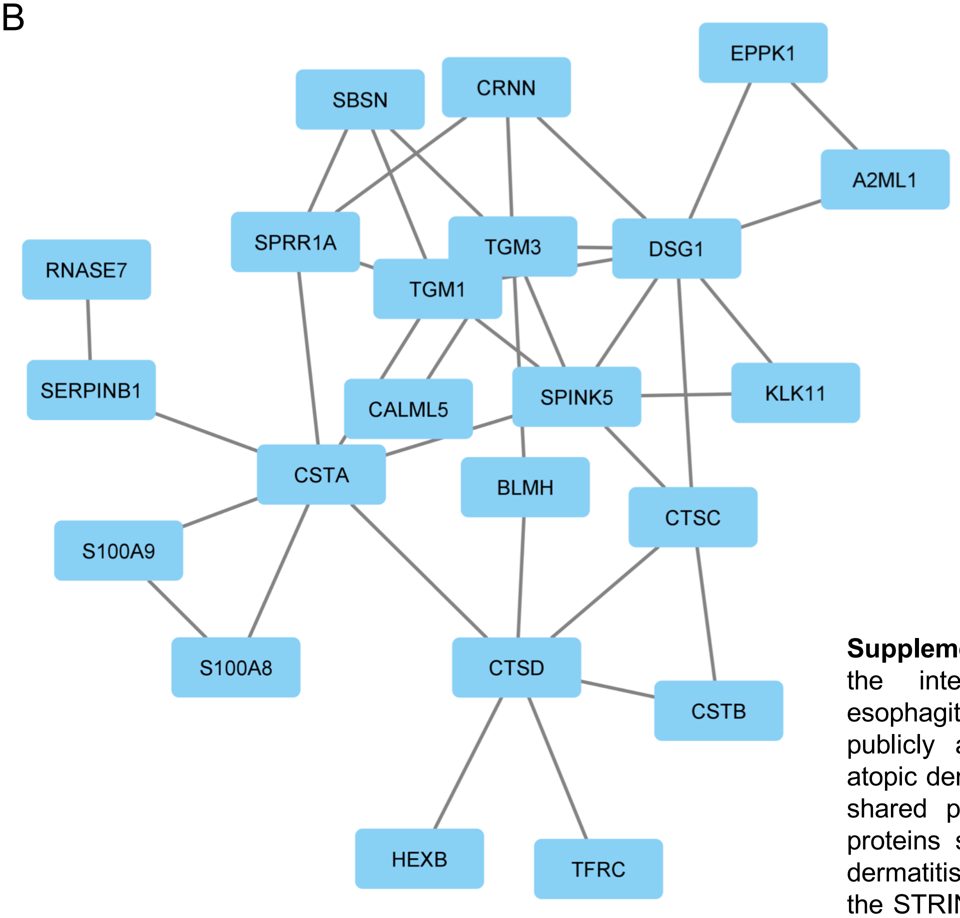

Supplementary Fig.2 A, Venn diagram of the intersection of the eosinophilic esophagitis (EoE) proteome with the publicly available proteome analyses of atopic dermatitis (31-34). The list shows all shared proteins. B, The connectome of proteins shared between EoE and atopic dermatitis identified in A was visualized by the STRING app in Cytoscape by applying a confidence score cut-off of 0.4 (described as “medium confidence”).
